# Prognosis and mortality within 90 days in community-acquired acute kidney injury in the Southwest of Sweden

**DOI:** 10.1186/s12882-023-03221-2

**Published:** 2023-06-13

**Authors:** Christel Gross, Junmei Miao Jonasson, David Buchebner, Björn Agvall

**Affiliations:** 1 Halland Hospital, Region Halland, Sweden; 2 Department of research and development, Region Halland, Halmstad, Sweden; 3 Department of Public Health and Community Medicine, University of Gothenburg, Gothenburg, Sweden

**Keywords:** Acute kidney injuries, Risk factors, Population register, Health resources, Prognosis, Chronic kidney disease, Mortality

## Abstract

**Background:**

Community-acquired acute kidney injury (CA-AKI) is common among hospitalized patients and has a poor prognosis. Research is scarce on the impact of a CA-AKI episode among patients without preexisting kidney disease and has not previously been investigated in Sweden. The aim was to describe the outcomes of patients with normal pre-hospitalization kidney function, admitted with community-acquired AKI and to investigate the association between AKI severity with outcomes.

**Methods:**

A retrospective population-based study was applied including patients with CA-AKI according to KDIGO classification, admitted via emergency department (ED) 2017–2019 and with a 90-day follow-up period from the ED-admission, collecting data from the Regional Healthcare Informative Platform. Age, gender and AKI stages, mortality and follow-up regarding recovery and readmission was registered. Hazard ratio (HR) and 95% confidence Interval (CI) for mortality was analyzed using Cox regression adjusted for age, comorbidities, and medication.

**Results:**

There were 1646 patients included, mean age was 77.5 years. CA-AKI stage 3 occurred in 51% of patients < 65 years of age and 34% among those > 65 years. In this study, 578 (35%) patients died and 233 (22%) recovered their kidney function. Mortality rate peaked within the first two weeks and among those at AKI stage 3. Nephrology referral post discharge occurred in 3% and 29% were readmitted. HRs for mortality was 1.9 (CI 1.38–2.62) for those who are > 65 years, 1.56 (CI 1.30–1.88) for atherosclerotic-cardiovascular disease. Medication with RAASi related to a decreased HR 0.27 (95% CI 0.22–0.33).

**Conclusions:**

CA-AKI is associated with high mortality within 90 days, increased risk for developing chronic kidney disease (CKD) and only one fifth recover their kidney function after hospitalization with an AKI. Nephrology referral was sparse. Patient follow-up after a hospitalization with AKI should be carefully planned during the first 90 days and focused on identifying those with a higher risk of developing CKD.

**Supplementary Information:**

The online version contains supplementary material available at 10.1186/s12882-023-03221-2.

## Introduction

Acute kidney injury (AKI) is a common consequence of acute illness that carries a substantially increased risk of mortality for many hospitalized patients [1–4]. Most survive to discharge and some even recover but there is growing evidence suggesting significantly increased long-term risk of chronic kidney disease (CKD), end-stage kidney disease, and death after an episode of AKI [5, 6].

Hospital-acquired AKI (HA-AKI) has been the focus of research over the last two decades. Less data has been published referring to the group of patients with previously normal kidney function, presenting at hospital with an existing acute kidney injury and defined as a community-acquired AKI (CA-AKI) [7–12]. CA-AKI represents the majority of AKI cases. According to several studies, CA-AKI incidence is 2 to 3 times higher than HA-AKI incidence and has the same prognostic significance as HA-AKI on mortality and higher healthcare costs [8, 9, 11, 13, 14].

The presence and severity of AKI predict mortality, bring with it the need of hospitalization, increased healthcare costs and a high prevalence of disease for both the individual and society [1, 6, 15, 16]. Mild AKI stage 1 is associated with a reduction in survival, which remains detectable for 10 years or more. Even patients attaining an apparent complete recovery remain at risk for long-term kidney complications [16–20].

CA-AKI is a serious condition that can occur acutely in previously kidney healthy individuals, but follow-up of kidney function is often limited. Management of concomitant diseases appears to be prioritized over CA-AKI in hospitalized patients [21–25]. AKI still is often dismissed as a benign event and patients have no acknowledgement of their AKI at hospital discharge [11, 21, 22]. Few patients have follow-up in primary healthcare (PHC) centers or nephrology clinic after discharge from hospital [10, 11, 21, 22].

While studies have demonstrated an association between CA-AKI and subsequent kidney complications, less is known about the prognostic implications of a hospital-associated CA-AKI episode in the subgroup of patients with normal pre-hospitalization kidney function, according to Kidney Disease: Improving Global Outcome (KDIGO) criteria [26].

The objective of the present study was to investigate prognosis and mortality in previously kidney-healthy individuals who visited the ED and simultaneously hospitalized with AKI. The aim was also to investigate the follow-up of this category of patient with an emphasis on their kidney function.

## Method

This is a retrospective, observational study of a population-based cohort admitted with AKI, according to KDIGO criteria, via emergency department (ED) to acute hospitals attending the residents of Region Halland [10].

The setting is Region Halland (RH), located in southwestern Sweden, and RH has an approximate population of 320,000 inhabitants. There are three hospitals, 40 inpatient wards, two EDs, 30 outpatient specialized clinics and 48 PHC facilities within RH.

### Data source

The study population is identified from the Regional Healthcare Information Platform (RHIP) in RH [27]. This database includes healthcare register data and offers a unique computerized ecological system that includes information from both primary and secondary healthcare levels. RHIP incorporates all prescribed and collected medications, clinical test results (i.e., laboratory assessments, radiological examinations) and healthcare resources utilized. All healthcare facilities, public and private use the same electronic healthcare record (EHR) system incorporated within the RHIP. Pharmacotherapy data was retrieved within RHIP through two sources: (i) the Swedish Prescribed Drugs Register and (ii) the pharmacy dos dispensing (Apodos).

### Study population

The inclusion criteria for the study were all individuals aged ≥ 18 years having a raised S-creatinine in connection with a visit to the ED, resulting in hospital admission. To be defined as CA- AKI, the patients had to have on admittance to the hospital or within 48 h of admission (index 0–48 h), a S-creatinine of ≥ 157 µmol/l for men and ≥ 135 µmol/l for women and a documented, regular S-creatinine was mandatory when reviewing the period of 7–365 days prior to index. The S-creatinine was considered as normal if ≤ 105 µmol/l for men and ≤ 90 µmol/l for women. The S-creatinine values from the lookback period defined the patient’s individual baseline S-creatinine. When the S-creatinine value was missing from the lookback period of 7-365 days prior the ED visit, that patient was excluded from the study. Included patients had to be a RH resident receiving care in RH at least one-year prior to the index visit to ED and during the study period after hospital discharge.

### Study procedure

The study period encompasses 2017 to 2019. After the individual visited the ED and was admitted to hospital, there was an observation period of 90 days.

The variables retrieved were gender, age at the time of the visit to the ED. The comorbidities were compiled from the lookback period 2013 until the index and the diagnoses defining the comorbidities according to ICD-10 which is displayed in Appendix–Table 2. CA-AKI was defined as occurring from any cause and did not separate the cases based on different etiologies. It was not possible to detect if the risk of progression to ‘de novo’ CKD or mortality differs amongst different causes of AKI. And, as the study was a retrospective cohort design, causal conclusions cannot be made.

S-creatinine (Scr) was collected from the lookback period prior to index, from the ED visit at index, peak S-creatinine during the hospital stay, at hospital discharge and from the follow-up period of 90 days after admission. Based on S-creatinine, the AKI stages were determined according to KDIGO. The definitions regarding the collection of S-creatinine in relation to time for the collection are further presented in Appendix - Table 1. Drug treatment regarding renin-angiotensin-aldosterone system inhibitors (RAASi) with ACT code C09, diuretics with ACT code C03 and non-steroid anti-inflammatory drugs (NSAID) with ACT code M01, the patient was on before the index-ED visit, were collected. The number of days the individual was hospitalized was recorded. The occurrence of readmission, dialysis and death, referral to nephrologist and follow-up with Scr-measurements during the 90-day study period were registered.

**Table 1:** Displays the baseline characteristics at index of the included patients in total and distributed by AKI stage at index admission

	Stage 1	Stage 2	Stage 3	Total	P-value
Total	381 (23)	677 (41)	588 (36)	1646 (100)	
*Gender*					
Women, n (%)	160 (42)	319 (47)	280 (48)	759 (46)	0.18^1^
Men, n (%)	221 (58)	358 (53)	308 (52)	887 (54)	
*Age groups*					
Age, mean (SD)	80.7 (10.9)	78.3 (11.2)	74.4 (11.8)	77.5 (12.6)	< 0.001^2^
18–64 years, n (%)	30 (8)	72 (11)	105 (18)	207 (13)	< 0.001^1^
≥ 65 years, n (%)	351 (92)	605 (89)	483 (82)	1439 (87)	
*Comorbidities*					
Hypertension, n (%)	268 (70)	452 (67)	348 (59)	1068 (65)	0.001^1^
ASCVD, n (%)	170 (45)	295 (44)	198 (34)	663 (40)	< 0.001^1^
Ischemic heart disease, n (%)	136 (36)	232 (34)	140 (24)	508 (31)	< 0.001^1^
Cerebrovascular disease, n (%)	36 (9)	55 (8)	48 (8)	139 (8)	0.72^1^
Peripheral vascular disease, n (%)	32 (8)	70 (10)	46 (8)	148 (9)	0.27^1^
Heart failure, n (%)	157 (41)	312 (46)	197 (34)	666 (41)	0.55^1^
Dementia, n (%)	36 (9)	65 (10)	46 (8)	147 (9)	0.50^1^
Diabetes, n (%)	103 (27)	200 (30)	183 (31)	486 (30)	0.40^1^
COPD, n (%)	67 (18)	164 (24)	134 (23)	365 (22)	0.04^1^
Connective tissue disease, n (%)	36 (6)	36 (5)	35 (6)	107 (6)	0.03^1^
Peptic ulcer, n (%)	19 (5)	41 (6)	52 (9)	112 (7)	0.04^1^
Liver disease, n (%)	0 (0)	10 (2)	25 (4)	35 (2)	0.77^1^
Tumor diseases, n (%)	113 (30)	235 (35)	190 (32)	538 (33)	0.24^1^
*Treatment at index*					
RAASi, n (%)	213 (56)	319 (47)	238 (40)	770 (47)	< 0.001^1^
Diuretics, n (%)	282 (74)	525 (78)	336 (62)	1173 (71)	< 0.001^1^
NSAID, n (%)	150 (39)	252 (37)	160 (27)	562 (34)	< 0.001^1^
*In-patient care at index*					
LOS, mean (SD)	6.0 (5.7)	6.3 (5.5)	7.7 (8.1)	6.7 (6.6)	< 0.001^2^
Days to readmission, mean (SD)	61 (36)	50 (38)	47 (38)	51 (38)	< 0.001^2^

Note; ASCVD = atherosclerotic cardiovascular disease, COPD = chronic obstructive pulmonary disease, RAASi = renin angiotensin aldosterone system inhibitor, NSAID = non steroid anti-inflammatory drug, LOS = length of stay at hospital admission. ^1^ Chi-2 test, ^2^ Kruskal Wallis test.

The primary outcomes were kidney function stratified by AKI stage 1–3 according to KDIGO criteria, and mortality. Secondary outcomes were hospital days, ICD code for kidney diagnosis and readmission.

There were two biochemistry laboratories, one at each hospital, which use Roche P-analyzer; Roche Diagnostics, Sweden covered all inpatient and outpatient samples, as well as all PHC practices. They provided all serum creatinine measurements throughout the study period (reference interval 45–90 µmol/l in women, 50–105 µmol/l in men).

### Statistical analysis

Descriptive analyses were performed for baseline characteristics variables. Continuous variables were described as means ± standard deviation (SD) and analyzed with Student-t test. Categorical variables were analyzed using Chi-2-Square tests and summarized using frequency and percentages. The incidence of CA-AKI for the year of 2017, 2018 and 2019 was calculated.

The population was distributed into two age groups, patients < 65 years of age and 65–85 years of age.

For adjustment of age, gender distribution, comorbidity and drug treatment, a cox regression analysis was performed. The Hazard ratio (HR) and the 95% confidence interval (CI) was used to estimate the relative risk of the occurrence of the outcome. The probability of survival during the follow-up was analyzed with a Kaplan- Meier curve.

All statistical tests were 2-sided and p < 0.05 identified significant differences. IBM SPSS Statistics 27.0 was used for statistical analysis.

## Results

In total, there were 1646 patients (54% men) included in the study. The incidence of CA-AKI during the three years 2017, 2018, and 2019 was stabile: 0.17%, 0.16% and 0.18%. The average age for the total cohort was 76.3 years (11.9) mean (SD) for men and 78.9 years (11.8) for women (p < 0.001). The distribution of gender, age groups, comorbidities, and medication in total and allocated to AKI stages are displayed in Table 1.

The mean age for each AKI stage and in total is shown in Table 1. The length of stay (LOS) of hospital admission from index was significantly longer in AKI stage 3. The number of days until readmission in average was significantly shorter in AKI Stage 3. LOS and days to readmission after discharge is shown in Table 1.

The kidney function in average at baseline, index and peak during hospital stay and during the follow up is as displayed in Table 2. The progress of the number of patients in the different AKI-stages from index across 90 days as shown in Table 2. After the 90-day study period, 330 (20% of the total cohort and 31% of the survivors) patients had an impairment of their residual kidney function showing persisting AKI Stage 1 to Stage 3 and fulfilled the KDIGO criteria for *de novo* CKD.

**Table 2:** Shows the patients’ kidney function and the progress regarding changes in AKI stages during the study period of 90 days

		AKI stages			
	Stage 1^1^	Stage 2^1^	Stage 3^1^	Total	P value
*Kidney function*					
Baseline Scr, mean (SD)	90 (16)	78 (30)	68 (17)	77 (16)	< 0.001
Index Scr, mean (SD)	160 (17)	180 (30)	302 (185)	219 128)	< 0.001
Maximal IPC Scr, mean (SD)	160 (18)	185 (36)	341 (203)	235 (148)	< 0.001
Discharge Scr, mean (SD)	117 (27)	123 (44)	178 (143)	143 (98)	< 0.001
30-days Scr, mean (SD)	122 (50)	118 (80)	142 (125)	128 (89)	0.001
90-days Scr, mean (SD)	126	114 (74)	120 (97)	118 (68)	0.30
*Kidney function within 90 days*					
AKI stage 0^2^, n (%)	61 (22)	100 (22)	72 (20)	233 (22)	< 0.001
AKI stage 1^2^, n (%)	53 (19)	117 (26)	79 (22)	249 (23)	
AKI stage 2^2^, n (%)	6 (2)	29 (6)	21 (6)	56 (5)	
AKI stage 3^2^, n (%)	2 (1)	3 (1)	20 (6)	25 (2)	
Missing value, n (%)	153 (40)	197 (29)	163 (27)	513 (31)	

Note; AKI = acute kidney injury, Scr = S-creatinine, IPC = inpatient care

^1.^ AKI severity staging definitions (Appendix - Table 1).

^2.^ AKI Stages within 90 days are AKI recovery stages (Appendix - Table 1).

**Table 3:** Mortality, frequency of dialysis, being admitted to ICU, nephrology referral, readmission within 90 days in total and by AKI stage at index

Outcomes, n (%)	Stage 1	Stage 2	Stage 3	Total	P value
Total cohort	381 (23)	677 (41)	588 (36)	1646 (100)	
Deaths	107 (28)	238 (35)	233 (40)	578 (35)	< 0.001
IPC deaths	36 (9)	104 (15)	126 (21)	266 (16)	< 0.001
Dialysis	1 (0)	2 (0)	18 (3)	21 (1)	< 0.001
Dialysis after discharged	1 (0)	0 (0)	18 (3)	19 (1)	< 0.001
ICU	37 (10)	105 (16)	167 (28)	309 (19)	< 0.001
Nephrologist referral	7 (2)	20 (3)	18 (3)	45 (3)	0.47
Readmission	93 (24)	211 (31)	172 (29)	476 (29)	0.25

Note; IPC = in-patient care, ICU = intensive care unit

Mortality, referral to nephrology, number of readmissions and distribution of AKI-stages after 90 days is shown in Table 3. A total of 578 (35%) died during the study period and of these, 266 (46% of the mortalities and 16% of the total cohort) patients died during the first hospitalization, without being discharged after the index. It was consequently 1380 (84%) of the total cohort who were discharged and among these there were 362 patients who had an ICD code that defined the impairment of kidney function.

**Fig. 1:**
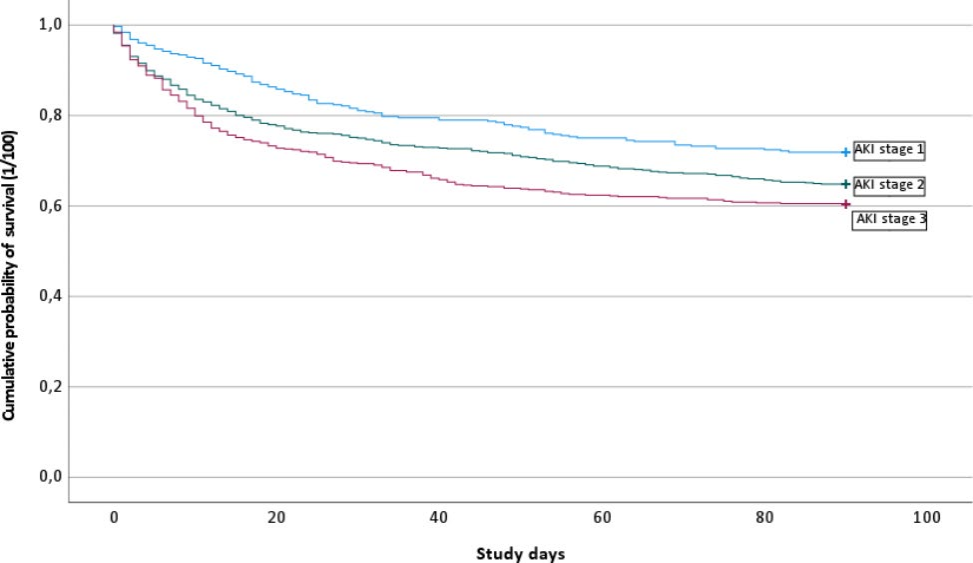
A Kaplan-Meier curve of the cumulative probability of survival for each AKI stage

Mortality was highest throughout the first 14 days and Fig. 1 shows how mortality stratified by AKI stages develops over time. Within 14 days after index, 311 (19%) patients died, and the 14-day mortality was highest in AKI Stage 3 in which 143 (24%) died. In AKI Stage 1 and AKI Stage 2 the 14-day mortality was 39 (10%) and 129 (19%) respectively.

The results of a cox-regression for mortality adjusted for gender, age, comorbidities, AKI severity and treatment are displayed in Table 4. An increased risk of death within the 90-day observation period associated with the older age group (> 65 years) [HR and 95% CI: 1.90 (1.38–2.629] when compared with the younger age group (< 65 years). The presence of certain chronic diseases, as atherosclerotic cardiovascular disease (ASCVD), related to an elevated risk of death as compared to those not having that disease [HR and 95% CI: 1.56 (1.30–1.88]. Similar results were found for dementia, heart failure and tumor diseases, respectively. Decreased risk of death was found for patients taking RAASi compared to those not taking RAASi with HR 95% CI: 0.27 (0.22–0.33).

**Table 4:** Adjusted Hazard Ratio for mortality and 95% CI from a Cox regression

	HR	95% CI for HR		p-value
		Lower	Upper	
*AKI Stage category*				0.02
AKI Stage 1	Reference			
AKI Stage 2	1.14	0.90	1.43	
AKI Stage 3	1.37	1.08	1.73	
Male gender	1.11	0.93	1.31	0.25
Age group ≥ 65 years	1.90	1.38	2.62	< 0.001
Diabetes	1.03	0.85	1.24	0.76
ASCVD	1.56	1.30	1.88	< 0.001
Dementia	1.59	1.24	2.03	< 0.001
COPD	1.22	1.01	1.48	0.04
Heart failure	1.91	1.56	2.33	< 0.001
Rheumatoid disease	0.82	0.57	1.18	0.29
Liver disease	1.72	1.11	2.65	0.01
Tumor diseases	1.43	1.20	1.71	< 0.001
RAASi	0.27	0.22	0.33	< 0.001
Diuretics	1.00	0.80	1.24	0.97
NSAID	0.69	0.57	0.85	0.001
Intensive Care Unit at index	1.40	1.14	1.71	0.001

Note; HR = Hazard ratio, ASCVD = atherosclerotic cardiovascular disease, COPD = chronic obstructive pulmonary disease, AKI = acute kidney injury, RAASi = renin angiotensin aldosterone system inhibitor, NSAID = non steroid anti-inflammatory drug

## Discussion

To the best of our knowledge, this is the first retrospective cohort study on the prognosis of *de novo* CA-AKI in Sweden. The individuals being hospitalized via ED and diagnosed with CA-AKI, showed a mortality rate of 35% within 90 days. Within the same period, 22% recovered to a normalized kidney function, but 30% had persisting kidney impairment, fulfilling the KDIGO criteria for *de novo* CKD. The factors influencing the Hazard ratio for mortality were age, ASCVD, dementia, COPD, HF, and tumor disease. Treatment with RAASi was associated with reduced mortality. Nephrology referral was sparse, and despite surviving and being discharged with persisting AKI, 31% did not have any Scr measurements taken within 90 days.

A high 90-day mortality rate of 35% in the present study was congruent with 41% in a comparative study [10]. Two other studies reported rates of 49% and 16.5%, respectively [10–12]. This disparity could be caused by different cohort definitions and different clinical settings. The study with the lowest mortality of 16,5% rate recruited the CA-AKI patients from hospital and the primary care setting, possibly gathering a younger and less severe diseased group meanwhile, higher mortality rates were associated with more patients having intensive care. [10–12]. Older age, cardiovascular diseases and tumor related to higher risk of death within 90 days among patients with CA-AKI which is consistent with other reports [1, 23]. The present study illustrated that age, heart failure, and diabetes are risk factors associated with worse prognosis after AKI, which is consistent with previous research [28, 29]. Moreover, it has been reported in earlier studies that patients with AKI stage 3 have a higher risk of developing CKD over time, and our findings align with these observations [30]. The prevalence of ASCVD was more common in AKI stage 1, which may appear irregular when comparing similar studies [14]. This unexpected correlation could be explained by the considerably higher mean age in AKI stage 1. In addition, the present study observed a higher mean age for the total cohort in comparison to the comparable study.

The study showed severe AKI to have a greater rate of in-hospital-mortality with 21% occurring in stage 3 as compared to 9% in stage 1. This graded relationship between mortality and severity of AKI is consistent with existing literature [14, 31].

One fifth (22%) of CA-AKI cases recovered in their kidney function within 90 days, while there were 30% of the study population with persisting AKI over a 90-day period, fulfillinged the criteria for *de novo* CKD according to KDIGO [28]. This is a lower rate compared with the 39.4% of *de novo* CKD reported in a previous study and might be due to a longer observation period [11].

As shown in previous studies, a significant proportion of patients, in spite of being alive, had no follow-up applying control Scr measurement in PHC and only 3% had a referral to nephrology after discharge from hospital with a rising creatinine. Out of 1380 discharged patients, just 362 (26%) had an ICD code defining impairment of kidney function. Unrecognized AKI is reported in various studies, although an association of nephrologist consultation with reduced adverse in-hospital renal and mortality outcomes in AKI patients has been shown [10, 11, 20–22]. Patients with a mild AKI (AKI Stage1) and those that even had a complete recovery of kidney function after an episode of AKI in subjects with normal baseline function, is associated with an increased risk of development of CKD [16, 18].

The benefit regarding mortality in those patients on regular prescription for RAASi is not surprising, because RAASi has a known nephroprotective effect and is therefore an indicated agent. In the present study, there were 56% of the patients treated with RAASi also shown in other comparable studies [20, 22, 32]. However, dosages were not collected nor if the intention to treat was kidney protection. Even so, RAASi seems to be associated with lower mortality. The present study had more treatment (78%) with diuretics compared to other studies and diuretics was not associated with lower mortality. Our study participants may have more prescriptions of diuretics and less prescriptions of RAASi compared to those in other studies [32, 33]. Statins have not been included in the analysis since there was a short follow-up period .

### Strengths and limitations

To avoid misclassification of underlying CKD as *de novo* CA-AKI, we used a rigorous definition for assessing *de novo* CA-AKI. This might come at the cost of missing out on those patients, who had never been tested prior to hospitalization or those entirely managed in primary care.

In our study, there was a higher mean value of S-creatinine in AKI stage 1 compared to AKI stage 2 and AKI stage 3. This can be explained by the fact that the number of men was higher in AKI stage 1 and also that the mean age was higher in this group.

In treatment with RAASi, diuretics or NSAID, dosage was not considered when retrieving the medication data but the presence of RAASi was shown to have an impact. The decrease of mortality regarding NSAID is difficult to explain. One reason could be, that patients with poor health do not receive prescriptions for NSAID.

Regarding prognosis and mortality, the findings in the present study should be generalizable to other regions in Sweden and even to other countries in the Western world. Furthermore, laboratory tests such as hemoglobin, albumin, electrolytes are variables not available in our study which might have contributed as prognostic markers. In this retrospective observational study, the results are associations, and it is not possible to have causal conclusions.

## Conclusions

CA-AKI is associated with a high mortality which was at its peak within the first two weeks of an AKI episode. Mortality risk is related to severity and duration of AKI-stage and is highest among patients with AKI stage 3. One fifth recover from AKI but an episode of AKI is associated with an increased risk for *de novo* CKD. Treatment with RAASi in AKI patients is associated with a lower Hazard ratio. Nephrology referral was sparse and follow-up among AKI patients after discharge should be carefully planned which could contribute to better outcomes.

## Electronic supplementary material

Below is the link to the electronic supplementary material.

Supplementary Material 1

## Data Availability

The datasets generated and analyzed in the current study are not publicly available due to the Swedish Health and Medical Services Act’s regarding the Secrecy Act but could be available from RH upon a reasonable request made to the corresponding author and followed by a specific review by the Regional Consultative Committee for data collection in RH.
