# Supplementary material for: Prognosis and mortality within 90 days in community-acquired acute kidney injury in the Southwest of Sweden
Source: BMC Nephrol. 2023 Jun 13;24:171. doi: 10.1186/s12882-023-03221-2 (PMC10262500; doi:10.1186/s12882-023-03221-2)
Supplement: Supplementary file 1 — Supplementary Material 1 [file 12882_2023_3221_MOESM1_ESM.docx]

**Supplementary**

Appendix-Table 1. De novo-COMMUNITY ACQUIRED AKI criteria definitions for study inclusion

| **Acute Kidney injury criteria for inclusion** | | |
| --- | --- | --- |
| *S-creatinine requirements*  a: S-creatinine ≥1,5 times higher than the serving laboratory’s upper reference interval 105 (x1,5=157) in men and 90 (x1,5=135) in women (= S-creatinine at admission or ≤48 hours of admission, referred to as ‘index creatinine’ and  b: documented S-creatinine value-/s 7-365 days prior to hospital admission, within the serving laboratory’s reference interval in µmol/l: 50-105 in men, 45-90 in women (lowest stable S-creatinine value is used), referred to as ‘baseline creatinine’. | | |
| *AKI severity staging definitions (maximum in-hospital S-creatinine):* | | |
|  | Stage 1 | Baseline ratio ≥1,5 - <2 |
|  | Stage 2 | Baseline ratio ≥ 2 - < 3 |
|  | Stage 3 | Baseline ratio ≥3 or on RRT |
| *AKI recovery status definition* | | |
|  | Complete recovery | S-creatinine <1,2 times higher than baseline S-creatinine |
|  | Partial recovery | S-creatinine >1,2 - <1,5 times higher than baseline S-creatinine |
|  | Nonrecovery | S-creatinine ≥ 1,5 times higher than baseline S-creatinine or RRT |

‘Untested’: no repeat blood tests taken at ‘discharge’/ ‘30-day’ and ‘90- day’ of ‘index creatinine’ (i.e., documented AKI onset); RRT=renal replacement therapy

Appendix–Table 2. Definitions for comorbidities according to specific ICD codes obtained from the period 2013 to 2019 and before index.

| **Comorbidity** | **ICD-10 codes** |
| --- | --- |
| Hypertension | I11.0, I13.0, I13.2 |
| Atherosclerotic cardiovascular disease | I20-25, I60-I66, G45, I693, I702, I739, I742-9 |
| Ischemic heart disease | I20-25 |
| Acute myocardial infarction | I21-I22, I25.2, I25.6 |
| Cerebrovascular disease | I60-I66, G45, I693 |
| Peripheral vascular disease | I702, I739, I742-9 |
| Heart failure | I42, I50 |
| Diabetes | E10-14 |
| Atrial fibrillation | I48 |
| Chronic obstructive pulmonary disease | J44 |
| Obesity | E66 |
| Rheumatoid disease | M300, M313-7, M329, M350-3 |
| Liver disease | K729 |
| Tumor disease | C00–99 |

Appendix–Table 3. Display the defined data collection of S-creatinine and its association to time period.

| **Defined data period** | **Definition of time** |
| --- | --- |
| Pre-index | 7-365 days prior to index |
| Index | 0-48 hours after ED-visit |
| Hospital peak value | Highest value during hospital stays |
| Discharge value | at discharge |
| Follow up period | Index until 90 days |
